# Efficient Utilization of Waste Carbon Source for Advanced Nitrogen Removal of Landfill Leachate

**DOI:** 10.1155/2017/2057035

**Published:** 2017-12-24

**Authors:** Kai Wang, Wenjun Yin, Fengxun Tan, Daoji Wu

**Affiliations:** School of Municipal and Environmental Engineering, Shandong Jianzhu University, Jinan 250101, China

## Abstract

A modified single sequencing batch reactor (SBR) was developed to remove the nitrogen of the real landfill leachate in this study. To take the full advantage of the SBR, stir phase was added before and after aeration, respectively. The new mechanism in this experiment could improve the removal of nitrogen efficiently by the utilization of carbon source in the raw leachate. This experiment adopts the SBR process to dispose of the real leachate, in which the COD and ammonia nitrogen concentrations were about 3800 mg/L and 1000 mg/L, respectively. Results showed that the removal rates of COD and total nitrogen were above 85% and 95%, respectively, and the effluent COD and total nitrogen were less than 500 mg/L and 40 mg/L under the condition of not adding any carbon source. Also, the specific nitrogen removal rate was 1.48 mgN/(h·gvss). In this process, polyhydroxyalkanoate (PHA) as a critical factor for the highly efficient nitrogen removal (>95%) was approved to be the primary carbon source in the sludge. Because most of the organic matter in raw water was used for denitrification, in the duration of this 160-day experiment, zero discharge of sludge was realized when the effluent suspended solids were 30–50 mg/L.

## 1. Introduction

Landfill leachate is produced from municipal waste deposited in the landfill. The leachate contains high concentrations of organics and ammonia nitrogen that are severe pollutants of the subsurface environment [1]. The efficient and cost-effective treatment of the leachate, aiming to meet discharge requirements, has become a worldwide interest. There is two kinds of technologies used for leachate treatments: physical/chemical treatment and biological treatment. The physical/chemical methods, such as ammonia stripping or advanced oxidation methods, are usually used for pretreatment or posttreatment because of their secondary pollution and high cost [2–4]. Biological treatments have been widely applied to treat landfill leachate because the reagents are reusable and cost-effective, and these procedures generate less secondary pollution [5–8].

The biological treatment of landfill leachate focuses on the removal of organics and ammonia nitrogen [9–14]. The organics are removed through conventional biological treatments, and the ammonia nitrogen is oxidized into NO_2_^−^-N or NO_3_^−^-N under aeration. Since the NO_2_^−^-N or NO_3_^−^-N is more poisonous than ammonia nitrogen, many countries (e.g., China) require that landfills meet total nitrogen (TN) emission standards on the landfill leachate. TN removal via denitrification requires a carbon source. However, most organics in the leachate have been oxidized into CO_2_ during aeration, resulting in a low concentration of carbon. The conventional biological treatments realize only 30%–70% nitrogen removal efficiency. For example, Laitinen et al. used sequencing batch reactor (SBR) activated sludge process combined with membrane bioreactor (MBR) to dispose of landfill leachate that contained 2200 mg/L chemical oxygen demand (COD) and 240 mg/L TN. The TN removal efficiency was below 60%. Yanjie et al. used granule sequencing batch reactors to dispose of landfill leachate, and the removal efficiency of TN was about 35.0%, with the ammonium of the leachate being 1105 mg/L. Mehdi used MBR to dispose of landfill leachate, and the removal efficiency of TN was about 28.0%. Some studies have reported high nitrogen removal in the leachate by adding an external carbon source, but this approach increased the cost and wasted the organics naturally present in the leachate [15].

Compared to other bioprocessing technologies, SBR has many advantages, such as simple structure and flexible operation. SBR has become a widely used method to dispose of landfill leachate [16]. Studies of SBR had focused on the removal of organics and ammonia, but little of them focused on the removal of total nitrogen.

Vock et al. discovered that activated sludge could store a carbon source, by transforming organic carbon from wastewater to the form of PHA stored in bacteria cells [17]. If the carbon-storage capability of the activated sludge could be strengthened, then TN removal would be able to use the stored indigenous carbon source, which would also improve the nitrogen removal efficiency and eliminate the addition of external sources of carbon [18].

To realize high nitrogen removal without the additional external sources of carbon, a modified SBR to treat real landfill leachate was used. The modified SBR differs from conventional SBR by adding a stirring phase before and after aeration phase, which allows for the full use of the carbon source in the leachate. Some parameters of SBR in one cycle which included COD, NH_4_^+^-N, NO_2_^−^-N, NO_3_^−^-N, TN, pH, and oxidation-reduction potential (ORP) were investigated. Also, the effect of variation of PHA of the sludge in one cycle of SBR was studied.

## 2. Materials and Methods

### 2.1. Landfill Leachate and Seed Sludge

Raw landfill leachate for this study was taken from the third MSW Sanitation Landfill Site, Jinan, Shandong province, China. The COD of the leachate was 3360 mg/L–4210 mg/L. The ammonia of the leachate was 860 mg/L–1012 mg/L, and NO_2_^−^-N and NO_3_^−^-N could be ignored. The characteristics of leachate were shown in Table 1. Inoculum was obtained from two sources: 80% of the inoculum is from the excess sludge of a pilot-scale SBR used to treat domestic wastewater, with MLSS and sludge volume index (SVI) being 10,000 mg/L and 350 mg/L, respectively; the rest of the inoculum (20%) came from an experimental SBR that had already produced nitridation with mature leachate, with MLSS and SVI being 3,500 mg/L and 56 mg/L, respectively. The MLSS, MLVSS, and SVI of the mixture were 8,523 mg/L, 6,375 mg/L, and 290 mg/L, respectively.

### 2.2. Experimental Setup and Operational Procedure

An SBR with working volume of 10 L was used in this study. The temperature was controlled at 25°C ± 1°C through the temperature control device, and the aeration was controlled through an air pump and gas flow meter.

**Figure 1:**
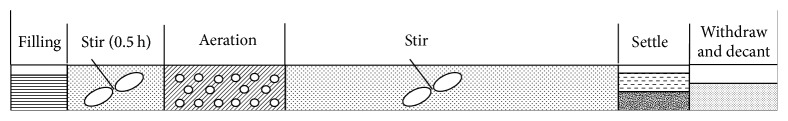
Operational mode of modified SBR.

The operation mode of the modified SBR is shown in Figure 1, including the filling phase, the anaerobic stir phase (synthesis of PHA in activated sludge), the aeration phase (nitration and simultaneous nitrification and denitrification), the anoxic phase (endogenesis denitrification), the settling phase, and the decanting phase, respectively. The dissolved oxygen (DO) concentration of nitritation was maintained in the range of 1.5–2.0 mg/L.

The sludge of the testing which investigated the content variation of PHA in one cycle was taken from the 120th experiment. In the testing, liquid samples and sludge samples were collected every 30 minutes before nitrification and every 60 minutes after nitrification. The liquid samples were analyzed for NH_4_^+^-N, NO_2_^−^-N, NO_3_^−^-N, TN, and COD. The sludge samples were analyzed for PHA.

During the experiment, COD, NH_4_^+^-N, NO_2_^−^-N NO_3_^−^-N, MLSS, and MLVSS were measured according to standard methods (APHA, 1998). The TN concentration was measured with Multi N/C3000 TOC (Analytik Jena AG, Germany). BOD_5_ was measured with an OxiTop Control WTW. DO, pH, and ORP were monitored using a pH/Oxi 340i analyzer (WTW Company, Germany). PHB was determined using the method of Zeng et al. [19].

## 3. Results and Discussion

### 3.1. Performance of the Modified SBR

#### 3.1.1. Quick Launch Strategy of Modified SBR to Realize Leachate Advanced Nitrogen Removal

The start-up phase lasted 35 d so that the inoculum had time to adapt to the characteristics of the leachate. The volumetric exchange rate (EVR) of SBR during this period was 10%.

**Figure 2:**
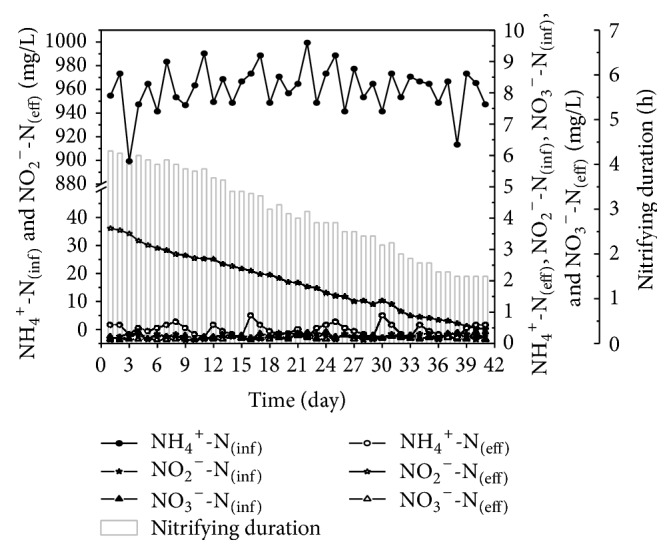
Fast start-up performance of modified SBR treating landfill leachate.

In order to enrich the quantity of the denitrifying bacteria, the operation time of anoxic stir stage was 10 hours. If TN was not removed in one cycle, external carbon sources (such as sodium acetate) were added to ensure the denitration finished. Since the initial SVI was high (290 mg/L), the settling time was set to 2 h to ensure the sludge would not flow away.  Figure 2 shows the performance of SBR during the start-up phase. The effluent NO_2_^−^-N concentration was measured at the completion of the anoxic stir stage without the addition of an external carbon source. The activity of the nitrite oxidizing bacteria (NOB) was inhibited because the free ammonia (FA) concentration in the leachate was 1.2918 mg/L (higher than domestic wastewater [0.775 mg/L]) and the DO concentration was low (i.e., 1.5–2.0 mg/L) during nitrification. Therefore, the system successfully achieved nitritation with a low production of NO_3_^−^-N (≤3 mg/L). During the previous 3 d of the experiment, the sludge did not adapt to the leachate, resulting in a system-specific ammonia oxidation rate (SAOR) of 3.55 mg NH_4_^+^-N/gvss·h and a nitritation stage that lasted 4 h. Moreover, there was about 35 mg/L NO_2_^−^-N remaining at the conclusion of the first cycle because of the small amount of denitrifying bacteria. Therefore, an external carbon source was added to remove more of the nitrogen.

After the ammonia oxidizing bacteria (AOB) adapted to the leachate, the nitritation stage decreased from 4.4 h (1st d) to 1.5 h on 38th d, while the SAOR increased to 9.64 mg NH_4_^+^-N/gvss·h and remained at about 10 mg NH_4_^+^-N/gvss·h for the rest of the experiment. The number of denitrifying bacteria grew rapidly in response to the addition of an external carbon source. On 41st d, the system achieved complete denitration without the addition of an external carbon source in one 14 h cycle, completing the start-up phase of the modified SBR.

#### 3.1.2. Ascension and Stability of Leachate Nitrogen Removal Performance of Modified SBR

**Figure 3:**
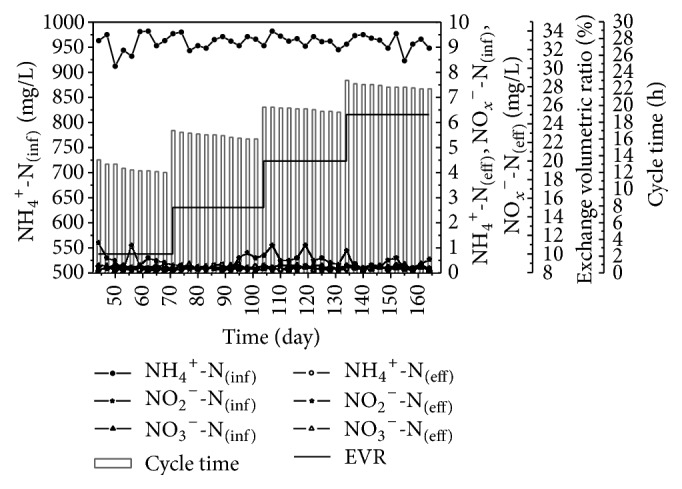
Performance of modified SBR treating landfill leachate with nitrogen load increasing.

After the completion of the start-up phase, the modified SBR system was able to efficiently remove nitrogen without adding external sources of carbon during any part of the cycle. As shown in Figure 3, the system operated for 30 d with the 10% EVR. The cycle completion time decreased from 13.4 h on 42nd d to 12 h on 70th d. Accordingly, the specific nitrogen removal rate (SNRR) increased from 0.99 mgN/h·gvss on 42nd d to 1.12 mgN/h·gvss on 70th d. After the cycle time was stable, the EVR was increased to enhance the denitrification capability of the sludge. On 71st d the EVR was increased to 15% and the influent TN of the system increased by 50%. However, the cycle time only increased to 17 h, suggesting an improvement in the denitrification efficiency.

Between 71st d and 101st d, the cycle time decreased from 17 h to 16 h and the SNRR increased to 1.29 mgN/h·gvss. Then we raised the EVR to 20% and the cycle time increased to 19.8 h. The cycle time did not improve proportionally with TN (the 20% EVR was only 65% longer than the 10% EVR and 23.7% longer than the 15% EVR). On 164th d we increased the EVR to 25% and the cycle time remained at 22 h with an SNRR of 1.48 mgN/h·gvss. The nitrogen removal efficiency of the 25% EVR increased by 30%, compared to the 10% EVR.

### 3.2. Detecting Reaction Progress with Parameter Variations

**Figure 4:**
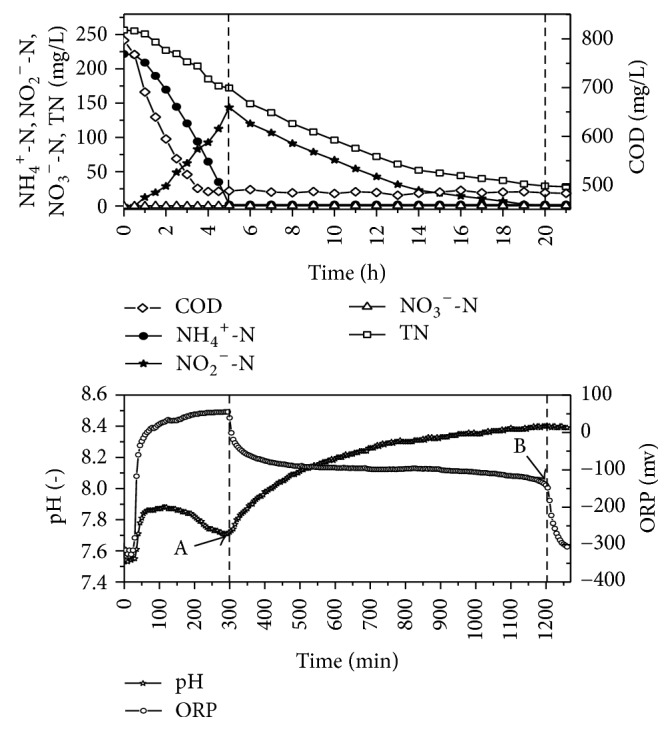
Key parameters variations of modified SBR in one cycle.

During the SBR reaction process, we could easily discern the conclusion of nitrification and denitrification stages by detecting changes in the system parameters, such as pH and ORP [20]. Variations of the parameters during one cycle of the modified SBR were shown in Figure 4. When the mixture contained an NH_4_^+^-N concentration of 225 mg/L after filling (EVR was 25%), the nitritation stage lasted 4.5 h. During the 4th h, a decrease in ammonia (Figure 4 point A) was easily discerned in the pH profile and signaled the end of nitritation. At this time, the aeration was stopped quickly in order to increase the denitration potentiality of SBR. During the last 3 h of nitritation, COD levels were greatly reduced, and after 3.5 h, COD remained at about 490 mg/L, indicating that the degradable organics had almost been entirely removed. During aeration, the levels of ammonia nitrogen decreased along with COD, which suggests high AOB activity. AOB acted as the dominant bacteria because there was no added nitrate. Furthermore, TN was also removed during aeration, as expected simultaneous nitrification and denitrification (SND). By the end of nitritation, TN concentration was reduced from 256 mg/L to172 mg/L, and the removal rate was 32.8%.

After the nitritation phase, the SBR system began to use the stored carbon source (such as PHA) to denitrify the leachate during a 15 h anoxic stage. During the 19th and 21st h of the whole reaction, a nitrate plateau (point B) was recorded in the ORP profile and suggested the end of denitration. At this time, NH_4_^+^-N and TN concentrations decreased below 5 mg/L and 30 mg/L, respectively. The nitrogen removal rate was above 95%. It is worth noting that there was no significant change in pH point during the anoxic stir stage, so the ORP was the only parameter which could be used to judge the terminal point of denitration.

During one cycle, about 2280 mg of nitrogen and 8540 mg of COD were removed. The total amount of COD used during denitration was above 4000 mg. At least 46.8% of the COD was consumed during denitrification. The modified SBR improved the utilization ratio of carbon and also reduced the aeration level in the leachate.

### 3.3. The PHA Variation of the Sludge in One Cycle

**Figure 5:**
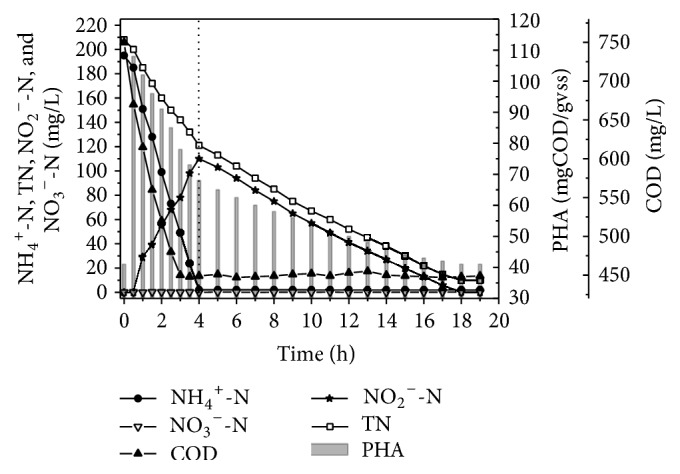
PHA variations of modified SBR in one cycle.

The system realized advanced nitrogen removal without adding any carbon source after aeration. It suggested that the activated sludge used its carbon source for denitrification. To understand the change of carbon sources during the denitrification process, on the 120th d of the test, the variation of PHA, NH_4_^+^-N, TN, NO_2_^−^-N, NO_3_^−^-N, and COD in one cycle was investigated. The test results are shown in Figure 5. According to the results of Figure 5, during the first half hour of the test, as the anaerobic mixing stage, the COD showed noticeable decline, while the PHA of sludge showed visible growth. This suggested that the organic matter of the wastewater was adsorbed by activated sludge and then was transformed to PHA. The PHA was the foundation of realizing advanced nitrogen removal. After half an hour, the system started to aerate, and the concentration of ammonia nitrogen and COD decline rapidly. At the same time, the PHA of the sludge was also reduced. Four hours later, the ammonia of the system was less than 5 mg/L which suggested the end of nitrification. At this point, the COD of the system was about 450 mg/L, and the removal rate of COD was about 85%. Because of the low DO concentration during the aeration, after the nitrification, the total nitrogen removal rate was about 40% by the simultaneous nitrification and denitrification (SND). The rest of the entire nitrogen mainly exists in the form of the nitrite. After aeration, the concentration of PHA decreased by about 40% in comparison with the very beginning of aeration, but compared to the beginging of the filling, the concentration of PHA increased significantly.

After the nitration, the system continues to stir. Due to the low dissolved oxygen of the system after stopping the aeration, the sludge began endogenous denitrification. During the process of endogenous denitrification, the concentration of TN and nitrate nitrogen steadily decreased and the PHA decreased synchronously. It is important to note that during the process of denitrification, the level of ammonia was not increased obviously, so we could deduce that the sludge used PHA for denitrification, rather than the carbon source from cell lyses. After 19 hours, the concentration of the nitrate nitrogen was nearly zero, and TN was about 20 mg/L which showed that advanced nitrogen removal had been completed. At this time, the concentration of PHA in sludge was about 40 mgCOD/GVSS which is equal to the concentration before filling.

According to this test, we knew that the concentration of PHA in sludge was the key point to realize advanced nitrogen removal. The anaerobic stir after filling provided the sludge opportunity to generate PHA which was the foundation of advanced nitrogen removal. The continue stir after nitration was the approach to realize advanced nitrogen removal. Under the joint action of efficient SND and reinforced endogenous denitrification, the system achieved advanced nitrogen removal without any external carbon source.

### 3.4. Sludge Variation during the Experiment

**Figure 6:**
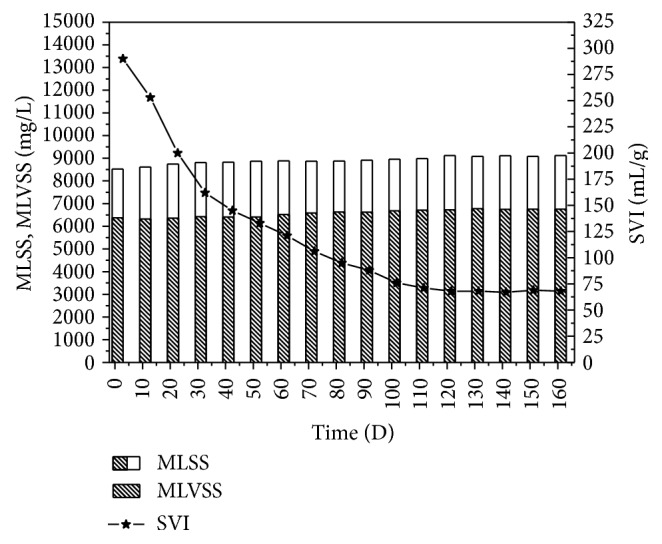
Variation of sludge during the experiment.

The activated sludge of modified SBR was fully adapted to the water quality of landfill leachate by more than one hundred days of cultivation and domestication. Variations of the MLSS, MLVSS, and SVI during the experiment were shown in Figure 6. At the beginning, the SVI value was 300 mL/g. The main reason was that the sludge contained a large number of filamentous bacteria. Since this experiment used alternative anaerobic/anoxic-aerobic modes, the growth of filamentous bacteria was inhibited. After 40 d, the SVI value of the sludge dropped to approximately 150 mL/g and then continued to reduce gradually, until eventual stabilization at about 65 ml/g. Since the sludge SVI value was high at the beginning of the test, we adopted tactics to prolong the precipitation time of the activated sludge. Denitrifying bacteria absorbed the vast majority of leachate organic matter, and there was limited growth of aerobic heterotrophic bacteria. In addition, the toxicity of leachate also inhibited the growth of sludge. After more than 160 experiment days, the growth speed of sludge in the system was slow, and there was zero discharge of sludge when the effluent SS was 30–50 mg/L.

## 4. Conclusions

This study modified the SBR operation mode by adding a stirring phase before and after the aeration stage. When the influent mixture had a COD of 3820 mg/L and a TN of 1000 mg/L, the modified SBR could achieve high COD removal (>85%) and extremely high total nitrogen removal (>95%) from real leachate without adding any external carbon source, and the maximum specific nitrogen removal rate reached 1.48 mgN/h·gvss.

In this study, the addition of stir before and after aeration was key element to achieve advanced nitrogen removal. The stir before aeration could cause the denitrifying bacterium to absorb carbon source and transfer it into an internal carbon source (e.g., PHA). The stir after nitrification could enable the denitrifying bacterium to realize endogenous denitrification through the utilization of internal carbon. Under the anaerobic/anoxic-aerobic operation mechanism, the utilization rate of organic matter (about 50%) for denitrification in wastewater was increased significantly. Because of that, during the 160 d experiment, the sludge concentration was maintained in the range of about 8000–9000 mg/L, and the system achieved sludge reduction dramatically.

## Figures and Tables

**Table 1:** The characteristics of the leachate.

Item	pH	NH_4_^+^-N (mg/L)	TN (mg/L)	NO_*x*_-N (mg/L)	COD (mg/L)	BOD_5_ (mg/L)
Range	7.8~8.2	860~1012	910~1106	0.11~0.8	3360~4210	1082~1380
Mean value	8.0	1010	1021	<1	3820	1215
